# Safety and Efficacy of Perioperative Intravenous Meloxicam for Moderate-to-Severe Pain Management in Total Knee Arthroplasty: A Randomized Clinical Trial

**DOI:** 10.1093/pm/pnab016

**Published:** 2021-01-27

**Authors:** Richard D Berkowitz, Richard Steinfeld, Alexander P Sah, Randall J Mack, Stewart W McCallum, Wei Du, Libby K Black, Alex Freyer, Erin Coyle

**Affiliations:** 1 University Orthopedic and Joint Replacement Center, Tamarac, Florida, USA; 2 Orthopaedic Center of Vero Beach, Vero Beach, Florida, USA; 3 Institute for Joint Restoration & Research, Fremont, California, USA; 4 Baudax Bio Inc., Malvern, Pennsylvania, USA; 5 Clinical Statistics Consulting, Blue Bell, Pennsylvania, USA

**Keywords:** Acute Pain, Health Economic Outcomes, Intravenous Meloxicam, Safety, Postsurgical Pain

## Abstract

**Objective:**

To evaluate the effect of perioperative meloxicam IV 30 mg on opioid consumption in primary total knee arthroplasty (TKA).

**Design:**

Multicenter, randomized, double-blind, placebo-controlled trial.

**Subjects:**

In total, 181 adults undergoing elective primary TKA.

**Methods:**

Subjects received meloxicam 30 mg or placebo via an IV bolus every 24 hours, the first dose administered prior to surgery as part of a multimodal pain management protocol. The primary efficacy parameter was total opioid use from end of surgery through 24 hours.

**Results:**

Meloxicam IV was associated with less opioid use versus placebo during the 24 hours after surgery (18.9 ± 1.32 vs 27.7 ± 1.37 mg IV morphine equivalent dose; *P *<* *0.001) and was superior to placebo on secondary endpoints, including summed pain intensity (first dose to 24 hours postdosing, first dose to first assisted ambulation, and first dose to discharge) and opioid use (48–72 hrs., 0–48 hrs., 0–72 hrs., hour 0 to end of treatment, and the first 24 hours after discharge). Adverse events (AEs) were reported for 69.9% and 92.0% of the meloxicam IV and placebo groups, respectively; the most common AEs were nausea (40% vs. 59%), vomiting (16% vs 22%), hypotension (14% vs 15%), pruritus (15% vs 11%), and constipation (11% vs 13%).

**Conclusions:**

Perioperative meloxicam IV 30 mg as part of a multimodal analgesic regimen for elective primary TKA reduced opioid consumption in the 24-hour period after surgery versus placebo and was associated with a lower incidence of AEs typically associated with opioid use.

**Key points:**

- **Question:** Does the perioperative administration of intravenous (IV) meloxicam reduce opioid consumption in the 24 hours after surgery in subjects undergoing primary total knee arthroplasty (TKA)?
- **Findings:** Perioperative meloxicam IV 30 mg was associated with 31.7% less opioid use versus placebo during the 24 hours after surgery (primary endpoint; 18.9 ± 1.32 vs 27.7 ± 1.37 mg IV morphine equivalent dose; *P* < .001). Opioid consumption at all other time intervals was also reduced, with statistically significant differences (*P* < .05) from placebo achieved for six out of seven comparisons (0–24 hours, 48–72 hours, 0–48 hours, 0–72 hours, 0–EOT, and 0–24 hours after discharge); the reduction at time 24–48 hours did not achieve statistical significance (*P* = .2549). Total charges related to hospital stays were approximately 10% lower for the meloxicam IV group compared with subjects in the placebo group. The duration of hospital stay was numerically shorter for subjects who received meloxicam IV versus placebo, but the difference was not statistically significant (*P* = .4935). Adverse events generally occurred in a lower percentage of the meloxicam IV group (69.9%) than of the placebo group (92.0%); there were no deaths or treatment discontinuations related to adverse events in either treatment group.
- **Meaning:** Perioperative administration of meloxicam IV 30 mg as part of a multimodal analgesic regimen in patients who underwent elective primary TKA demonstrated a reduction in opioid consumption in the 24 hours after surgery and was associated with reductions in certain healthcare resource utilization measures. Adverse events occurring at a higher rate in meloxicam IV 30 mg versus placebo included: pruritus (15% vs 11%), pyrexia (8% vs 6%), anemia (3% vs 2%), tachycardia (3% vs 1%), and urinary retention (3% vs 1%), with all other AEs occurring at a similar or lower rate compared with placebo. These results suggest a promising role for meloxicam IV as part of a multimodal analgesic regimen in this clinical setting.

## Introduction

Patients typically report high levels of pain after orthopedic surgery, and managing this pain can be challenging [1–3]. The importance of effective pain management is underscored by adverse outcomes associated with uncontrolled postoperative pain, which include delayed recovery, longer hospital stays, readmissions, increased morbidity, and decreased quality of life [1, 3, 4]. In an effort to improve the management of postoperative pain, current guidelines strongly recommend an integrative approach, including use of multimodal analgesia and minimization of opioids [3, 5, 6].

In the past, the limited number of options to treat moderate-to-severe pain led to overreliance on the use of opioids [7]. Given the potential risks associated with opioids, such as addiction, gastrointestinal adverse events (AEs), pruritus, and respiratory depression, among others, there has been an increased emphasis on using alternative medications and decreasing opioid use in patients who undergo elective or nonelective surgical procedures [5, 8–12]. In general, it is recommended that opioids be used at the lowest possible dose and not in isolation [8, 9]. Therefore, a multimodal analgesic regimen that includes two or more analgesic agents with different mechanisms of action to provide enhanced analgesia is a rational approach [5]. Nonsteroidal anti-inflammatory drugs (NSAIDs) are recommended for this purpose, as they have not only demonstrated efficacy for reducing postoperative pain, but have also demonstrated benefits with respect to patient satisfaction (improved), opioid consumption and related AEs (reduced), systemic inflammation (reduced), and time to recovery (shortened) [5, 13–15]. However, they can lead to serious side effects, including gastrointestinal bleeding, exacerbation of respiratory disease, thrombotic events, and renal or hepatic injury [16, 17], the risk of which may be related to cyclooxygenase-2 (COX-2) selectivity. Furthermore, few intravenous (IV) options are currently available.

Meloxicam is an NSAID with a prolonged half-life that has preferential, but not exclusive, inhibition of COX-2, demonstrating a more favorable gastrointestinal AE profile (ie, lower rates of GI-related AEs [dyspepsia, nausea/vomiting, abdominal pain, diarrhea, GI mucosal damage]) than that of nonselective NSAIDs [18]. Intravenously administered meloxicam (meloxicam IV) utilizes a novel nanocrystal formulation of meloxicam and has been evaluated in seven phase 2 and 3 postoperative studies in subjects with moderate-to-severe pain following hard-tissue surgery [19–22] or soft-tissue surgery [21, 23–25].

The primary objective of this study was to evaluate the effect of perioperative administration of meloxicam IV 30 mg (first dose administered prior to surgery) on opioid consumption in subjects undergoing primary total knee arthroplasty (TKA). In this study, meloxicam IV was used as part of a multimodal pain management regimen to provide pain relief in accordance with generally accepted good practices [2, 3, 5]. Secondary objectives were to assess the safety of meloxicam IV 30 mg and to evaluate its effect on postoperative pain, as well as associated health care resource utilization, compared with that of placebo.

## Methods

### Study Design and Subjects

This was a randomized, double-blind, placebo-controlled, multicenter study in adult subjects undergoing elective unilateral TKA; it was conducted in hospital settings and was planned to require a hospital stay of ≥24 hours. Subjects were randomized (1:1) to receive meloxicam IV 30 mg or placebo IV as a bolus injection every 24 hours, consistent with meloxicam dosing recommendations. The randomization scheme was generated prior to study initiation by an independent statistician who was not involved in the study. Randomization was assigned by central Interactive Web Response Systems (IWRS), with access limited to unblinded personnel. Matching placebo was utilized to prevent unblinding. Study medication was administered in addition to a multimodal pain management protocol that included perioperative use of analgesics with differing mechanisms of action. The study was designed and monitored in accordance with the ethical principles of Good Clinical Practice and in accordance with the Declaration of Helsinki. The protocol was approved by a central Institutional Review Board (Western Institutional Review Board Protocol 20172394) and the local institutional review boards of participating institutions when required. All subjects provided written informed consent. The trial was registered prior to patient enrollment at clinicaltrials.gov (NCT03434275; Date of registration: February 15, 2018). Each study site had its own principal investigator (Supplemental Digital Content 4, Table S3).

Eligible subjects were males and females, aged 35–80 years (inclusive), who were planning to undergo elective primary (ie, no repeat procedures) unilateral TKA surgery. Participants were expected to require IV analgesia, remain in an inpatient setting for ≥24 hours, and expected but not required to receive ≥2 doses of study drug. Subjects were also required to be nonpregnant, to use effective contraception, and to have an American Society of Anesthesiology (ASA) physical status ≤3 (normal healthy patients, patients with mild systemic disease, or patients with severe systemic disease [26]), a body mass index ≤40 kg/m^2^, and a performance status that allowed the subject to carry on normal activities of daily life without limitations.

Excluded from participation were subjects with a history of previous TKA, those with plans for a concurrent surgical procedure (eg, bilateral TKA), and those undergoing unicompartmental knee replacement or revision TKA. Other exclusion criteria included a diagnosis of rheumatoid or inflammatory arthritis or related systemic inflammatory disease; a myocardial infarction within 12 months; significant renal, hepatic, cardiovascular, metabolic, neurologic, psychiatric, or respiratory disease; or clinically significant abnormal clinical laboratory values. Other reasons for exclusion were gastrointestinal ulceration or bleeding within 6 months, a known bleeding disorder, a clinically significant 12-lead electrocardiogram abnormality, long-term use of opioid therapy (ie, daily for ≥30 days), >50 days of opioid use within 30 days before screening, or use of long-acting opioids within 3 days of the surgical procedure. Disallowed medications included NSAIDs (within 48 hours of surgery), herbal medications/supplements associated with increased bleeding risk (eg, ginkgo biloba, garlic, ginger, ginseng, hawthorn, fish oil, dong quai, feverfew, vitamin E) within 7 days. Subjects receiving lithium, or furosemide plus either an angiotensin-converting enzyme inhibitor or angiotensin receptor blocker were also excluded due to the potential for drug-drug interactions between NSAIDs and these agents [27].

### Study Drug Administration

Subjects received either meloxicam IV 30 mg or placebo IV prior to surgical incision, then once daily while in the hospital, until discharge or until IV analgesia was no longer appropriate. Subjects also received a standardized clinical care protocol. This included venous thromboembolism prophylaxis (administered before and after surgery according to standard practice), based on the subject’s individual needs, at the discretion of the investigator and surgeon. Study medication was combined with a multimodal analgesic protocol for pain management. As part of multimodal pain management, subjects received oral acetaminophen (650 mg) and oral gabapentin (600 mg) administered 30 to 90 minutes before surgery. Other concomitant medication included prophylactic IV antibiotic and tranexamic acid (1 g IV) 30 to 90 minutes prior to surgery and at the end of surgery, and IV ondansetron (4 mg) as needed for treatment of postoperative nausea and vomiting. The study drug was administered following spinal anesthesia and before the start of surgery (ie, time of incision). Immediately before wound closure, subjects received local infiltration of the surgical site with bupivacaine hydrochloride (0.5% 30 mL) and epinephrine (5 μg/mL), expanded in a volume of 90 mL of normal saline.

The end of the surgical procedure (hour 0) was defined as the time of last suture, staple, or steri-strip. Subjects had access to IV and oral opioid medication, as needed, for the management of breakthrough pain, beginning at hour 0 and continuing until discharge. Postoperative opioid pain medications were administered per subjects’ request and included 1–4 mg of morphine IV up to every 10 minutes for the first hour, then 1–8 mg IV up to every hour, as needed, converting to oral immediate-release oxycodone 5 mg every 4 hours, as needed (maximum of 10 mg every 4 hours), once liquid intake was tolerated. All subjects received oral acetaminophen, 650 mg every 8 hours (as tolerated), until 24 hours following the last dose of study drug. No other analgesic agents were allowed except aspirin for venous thromboembolism prophylaxis.

### Endpoints

The primary efficacy parameter was the total use of opioid analgesia from end of surgery (EOS; hour 0) through 24 hours. To analyze total opioid consumption, medication use was converted to IV morphine equivalent dose (IVMED, mg) using a standardized conversion table.

Pain intensity (PI) was assessed using an 11-point numeric pain rating scale (0–10; 0 = no pain, 10 = worst imaginable pain) at defined intervals throughout the first 48 hours after surgery, before and during each ambulation, and before each administration of opioid rescue (time 0 through hospital discharge). The sum of PI score (SPI) was the time-weighted cumulative PI value from first dose [28]. The weight factor at each time point was the time elapsed since the previous observation [29]. The SPI is also referred to as the SPID when the baseline pain score is nonzero. In this study, all subjects had baseline postsurgery pain score of zero because the study medication was given before surgery. Outcome measures that summarize treatment response over a clinically relevant period are widely reported in clinical trials of analgesics [30–33]. Secondary endpoints included the sum of PI from the time of first dose of study drug through 24 hours, the percentage of opioid-free subjects from EOS through 24 hours, and the time from EOS to the first use of IV opioid as rescue medication. Other efficacy endpoints included the sum of PI over other time intervals, the percentage of subjects who were opioid free over other time intervals, the total use of opioid analgesia over other intervals, and the 7-item patient-reported Overall Benefit of Analgesia Score (OBAS) questionnaire.

Safety endpoints included the incidence of AEs, clinically significant clinical laboratory values and vital signs, and investigator satisfaction with wound healing before hospital discharge and during the follow-up visit (which occurred on postoperative days 10–14). AEs of special interest included selected events related to concerns associated with NSAIDs: bleeding, injection-site reactions, and cardiovascular, hepatic, renal, thrombotic, and wound healing events. Events commonly associated with opioid administration were also tabulated, including gastrointestinal (nausea, vomiting, constipation, stomach pain, loss of appetite, ileus), central nervous system (sleepiness, tiredness, drowsiness, dizziness, light-headedness, weakness, itching, dry mouth), and respiratory effects (respiratory depression, apnea, respiratory arrest) [34].

Also analyzed was health care resource utilization. These additional assessments included hospital length of stay, hospital readmission, total hospital charges, postsurgical physical therapy visits, emergency department visits, use of skilled nursing facility, and phone calls related to postsurgical pain.

Telephone interviews were conducted 24 and 48 hours after hospital discharge by the investigator or a qualified designee to assess opioid medication use, PI, and health care resource utilization. A postoperative clinical follow-up visit occurred between postoperative days 10 and 14, and the final follow-up telephone interview was conducted on postoperative day 30.

### Statistical Analysis

The anticipated sample size of the study (100 subjects per group) had a ≥ 90% power to detect the difference between meloxicam IV 30 mg and placebo in total opioid consumption, based on the results of the phase 3 safety study that evaluated meloxicam IV 30 mg in major surgeries [21]. Results are reported as mean values ± standard error (SE). Treatment effect analyses were performed on the modified intent-to-treat analysis set, which included all subjects who received ≥1 injection of study drug and underwent the scheduled surgery. Treatment effect was evaluated using analysis of covariance (ANCOVA) for opioid consumption‒related endpoints and PI-related endpoints. The ANCOVA model included treatment as the main effect and investigational site as a covariate. Differences in least squares (LS) means were compared between the treatment groups. Differences between meloxicam IV 30 mg and placebo groups were evaluated via a 2-sided, 2-sample *t*-test at the 0.05 significance level. Kaplan-Meier survival analysis was performed for time-to-event endpoints, including Kaplan-Meier survival curves; 25th-, 50th-, and 75th-percentile estimates; and corresponding 95% confidence intervals (CIs). The magnitude of treatment effect of meloxicam IV 30 mg versus placebo for time-to-event endpoints was analyzed using a Cox proportional hazards (PH) model to estimate hazard ratios (HR; meloxicam IV 30 mg/placebo). The Cox PH model included treatment effects and investigational sites. HR estimates and 95% CIs were based on Wald’s statistics.

Safety and tolerability assessments were performed on the safety set, which included all treated subjects. Safety endpoints and health care utilization measures were analyzed descriptively.

## Results

**Figure 1.**
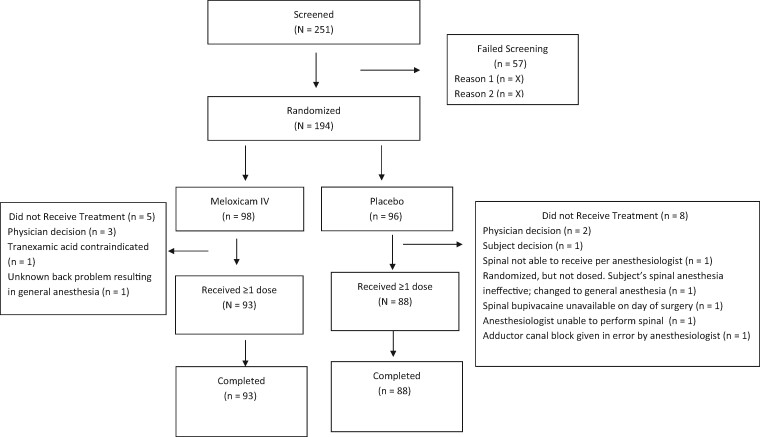
Subject disposition.

A total of 251 subjects were screened, 194 of whom were deemed eligible and assigned randomly to a study group. Of these, 181 (meloxicam IV, *n* = 93; placebo, *n* = 88) received ≥1 dose of study drug, and all treated subjects completed the study through the last visit (Figure 1). Baseline demographics, and surgical characteristics are summarized in Table 1. In general, study groups were similar in terms of demographic and surgical characteristics. Most surgeries (69.1%) were able to spare or minimize invasion of the quadriceps tendon. The majority of subjects in both groups received 2 or 3 doses of study drug (Table 2).

**Table 1.** Subject disposition, demographics, and surgical characteristics

	Meloxicam IV 30 mg	Placebo	Overall
	(*n* = 93)	(*n* = 88)	(*N* = 181)
Mean age, y (SD)	66.9 (8.2)	65.5 (8.1)	66.2 (8.2)
Age ≥65 y, n (%)	58 (62.4)	54 (61.4)	112 (61.9)
Female, n (%)	54 (58.1)	51 (58.0)	105 (58.0)
Race, n (%)			
White	74 (79.6)	70 (79.5)	144 (79.6)
Black or African American	18 (19.4)	17 (19.3)	35 (19.3)
Asian	1 (1.1)	1 (1.1)	2 (1.1)
Ethnicity, n (%)			
Hispanic or Latino	9 (9.7)	9 (10.2)	18 (9.9)
Not Hispanic or Latino	84 (90.3)	79 (89.8)	163 (90.1)
Mean baseline BMI, kg/m^2^ (SD)	30.6 (4.7)	31.5 (5.1)	31.1 (4.9)
Surgery knee, n (%)			
Left	56 (60.2)	38 (43.2)	94 (51.9)
Right	37 (39.8)	50 (56.8)	87 (48.1)
Quadriceps tendon spared, n (%)	67 (72.0)	58 (65.9)	125 (69.1)
Mean (SD) surgery duration, h	1.3 (0.22)	1.3 (0.24)	1.3 (0.23)
Mean (SD) time in PACU, min	94.8 (57.2)	87.4 (45.0)	91.2 (51.6)
Post PACU disposition, n (%)			
Step-down unit	23 (24.7)	22 (25.0)	45 (24.9)
General medical/surgical	70 (75.3)	65 (73.9)	135 (74.6)
Other	0	1 (1.1)	1 (0.6)

BMI = body mass index; IV = intravenous; PACU = post anesthesia care unit; SD = standard deviation.

**Table 2.** Study drug exposure

	Meloxicam IV 30 mg	Placebo
Number of doses, n (%)	(n = 93)	(n = 88)
1	1 (1.1)	6 (6.8)
2	32 (34.4)	28 (31.8)
3	49 (52.7)	38 (43.2)
4	11 (11.8)	16 (18.2)

### Efficacy

With respect to the primary endpoint (opioid consumption in the first 24 hours after EOS), opioid use was significantly lower with meloxicam IV-treated subjects (18.9 mg ± 1.32 IVMED vs 27.7 mg ± 1.37 IVMED; *P *<* *.0001), corresponding to 31.7% less opioid usage during this period (Supplemental Digital Content 1, Figure S1). Meloxicam IV was also associated with statistically significant differences from placebo for several secondary endpoints. For example, opioid consumption was lower in the meloxicam IV group at all other time intervals, with statistically significant differences from placebo for most comparisons (Supplemental Digital Content 2, Table S1). Opioid usage was numerically lower for subjects in the meloxicam IV group during hours 24–48 (9.7% decrease; *P *=* *.2449) and significantly lower for subjects in the meloxicam IV group during hours 48–72 (32.1% decrease; *P *=* *.0306), hours 0–48 (24.3% decrease; *P *<* *.0001), hours 0–72 (25.0% decrease; *P *<* *.0001), hour 0 to end of treatment (25.9% decrease; *P *=* *.0002), and in the first 24 hours after discharge (19.1% decrease; *P *=* .*00394).

**Figure 2.**
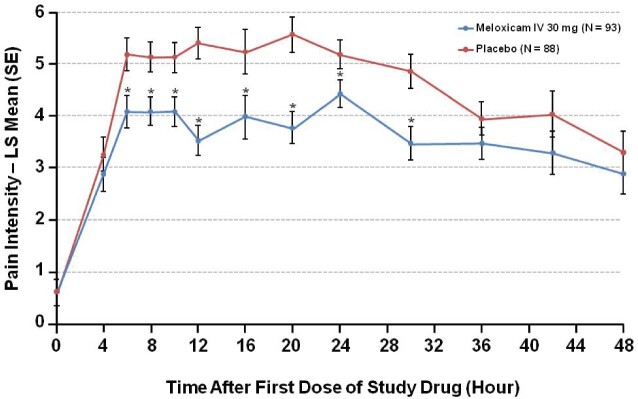
Pain intensity—observed. LS = least squares; SE = standard error.

At multiple time points during the initial 24 hours postdose, PI was lower in subjects treated with meloxicam IV (Figure 2). Moreover, summed PI scores were better for subjects who received meloxicam IV versus placebo for most time intervals, including notable recovery milestones (eg, first dose to 24 hours postdosing [*P *<* *.0001], first dose to first assisted ambulation [*P *=* *.0235], and first dose to discharge [*P *=* *.0001] [Table 3]). The OBAS score was significantly lower for meloxicam IV compared with placebo-treated subjects on the first postoperative day (LS mean [SE] 4.45 [0.360] vs 5.90 [0.375] for meloxicam and placebo, respectively; difference [95% CI], –1.45 [–2.39, –0.51]; *P *=* *.0027). Changes in OBAS over other intervals are summarized in Table 4.

**Table 3.** Sum of time-weighted pain intensity*

	Meloxicam IV 30 mg	Placebo	*P*-value
Parameter	(*n* = 93)	(*n* = 88)	
From first dose until*:* 24 hours after dosing			
LS mean (SE)	5328 (238.1)	6854 (248.6)	< .0001
First assisted ambulation			
LS mean (SE)	2211 (355.4)	3307 (378.6)	.0235
First independent ambulation			
LS mean (SE)	5170 (1566)	8151 (2262)	.3018
Discharge			
LS mean (SE)	10,541 (993)	15,670 (1045)	.0001

* The sum of PI score (SPI) was the time-weighted cumulative PI from first dose [28]. The weight factor at each time point was the time elapsed since the previous observation. The SPI is also referred to as the SPID when the baseline pain score is nonzero. In this study, all subjects had baseline postsurgery pain score of zero because the study medication was given before surgery. LS = least squares; SE = standard error.

**Table 4.** Overall benefit of analgesia score (OBAS)

Parameter	Meloxicam IV 30 mg	Placebo	*P*-value
Postoperative day 1	n = 92	n = 84	
LS mean (SE)	4.45 (0.360)	5.90 (0.375)	.0027
Postoperative day 2	n = 76	n = 65	
LS mean (SE)	3.85 (0.379)	4.49 (0.400)	.1803
Postoperative day 3	n = 12	n = 21	
LS mean (SE)	3.89 (0.805)	3.95 (0.705)	.9524
Prior to discharge	n = 92	n = 84	
LS mean (SE)	3.82 (0.322)	4.51 (0.336)	.1054

Following surgery, subjects were able to request opioid analgesia as needed (ie, rescue medication). Time from EOS to first use of opioid rescue medication (via IV or oral administration) was significantly longer with meloxicam IV versus placebo (*P *=* *.0003) (Table 4). Meloxicam IV was associated with a longer time to first IV rescue medication (*P *=* *.0009) and a longer time to first oral rescue medication (*P *=* *.0036) (Table 5).

**Table 5.** Time (hours) from end of surgery to first rescue medication

Statistics	Meloxicam IV 30 mg	Placebo
	(n = 93)	(n = 88)
Opioid rescue (via IV or oral administration)		
Subjects censored, n (%)	1 (1.1)	0
25th percentile KM estimate (95% CI), h	2.20 (1.13, 2.65)	1.06 (0.57, 1.80)
50th percentile KM estimate (95% CI), h	3.38 (3.10, 3.97)	2.78 (2.23, 3.28)
75th percentile KM estimate (95% CI), h	5.30 (4.17, 6.77)	4.08 (3.57, 4.97)
KM mean (SE) time, h	4.94 (0.54)^a^	3.09 (0.28)
HR estimate (95% CI)	0.559 (0.409, 0.763)	
Wald’s χ^2^ test *P* value	.0003	
Opioid rescue (via IV administration)		
Subjects with event, n (%)	30 (32.3)	11 (12.5)
25th percentile KM estimate (95% CI), h	2.65 (1.47, 4.42)	1.11 (0.57, 1.97)
50th percentile KM estimate (95% CI), h	6.22 (4.83, 8.15)	3.65 (2.95, 5.53)
75th percentile KM estimate (95% CI), h	18.25 (9.40, 24.70)	6.77 (5.90, 10.88)
KM mean (SE) time, h	10.85 (1.38)^a^	6.16 (0.83)
HR estimate (95% CI)	0.555 (0.393, 0.786)	
Wald’s χ^2^ test *P* value	.0009	
Opioid rescue (via oral administration)		
Subjects with event, n (%)	1 (1.1)	0
25th percentile KM estimate (95% CI), h	3.28 (2.75, 3.75)	2.70 (2.40, 3.07)
50th percentile KM estimate (95% CI), h	4.28 (3.88, 5.60)	3.94 (3.30, 4.38)
75th percentile KM estimate (95% CI), h	8.12 (6.52, 12.05)	5.28 (4.68, 6.17)
KM mean (SE) time, h	7.69 (0.85)^a^	5.22 (0.52)
HR estimate (95% CI)	0.636 (0.469, 0.863)	
Wald’s χ^2^ test *P* value	.0036	

CI = confidence interval; h = hour; HR = hazard ratio; KM = Kaplan-Meier; SE = standard error.

a The KM mean time to event may have been underestimated because the largest observation was censored, and the estimation was restricted to the largest event time.

### Adverse Events

The majority of study subjects (146/181 [80.7%]) experienced ≥1 AE, but the overall incidence of subjects with an AE was lower in the meloxicam IV group than in the placebo group (69.9% vs 92.0%) (Table 5). There were no AE-related treatment discontinuations or deaths in either study group. The most commonly reported AEs were nausea, vomiting, hypotension, pruritus, and constipation (Table 6). The majority of events in the meloxicam IV group were of mild (79%) or moderate (20%) intensity. Fewer subjects reported serious AEs (SAEs) in the meloxicam IV group (3 vs 9). The SAEs in the meloxicam IV group were anemia, rectal hemorrhage, and syncope (1 each); SAEs in the placebo group were 2 instances of pulmonary embolism and 1 each of atrial fibrillation, congestive cardiac failure, esophageal stenosis, cellulitis, hyponatremia, transient ischemic attack, acute kidney injury, hypertension, and deep vein thrombosis.

**Table 6.** Adverse events that occurred in ≥3 subjects.

	Meloxicam IV 30 mg	Placebo
Preferred term, n (%)	(n = 93)	(n = 88)
Subjects with ≥1 AE	65 (69.9)	81 (92.0)
Subjects with ≥1 serious AE	3 (3.2)	9 (10.2)
Nausea	37 (39.8)	52 (59.1)
Vomiting	15 (16.1)	19 (21.6)
Hypotension	13 (14.0)	13 (14.8)
Pruritus	14 (15.1)	10 (11.4)
Constipation	10 (10.8)	11 (12.5)
Dizziness	6 (6.5)	5 (5.7)
Pyrexia	7 (7.5)	5 (5.7)
Hypokalemia	2 (2.2)	6 (6.8)
Hypertension	0	7 (8.0)
Headache	1 (1.1)	5 (5.7)
Insomnia	3 (3.2)	3 (3.4)
Anemia	3 (3.2)	2 (2.3)
Tachycardia	3 (3.2)	1 (1.1)
Urinary retention	3 (3.2)	1 (1.1)
Cellulitis	0	3 (3.4)
Rash	0	3 (3.4)

AE = adverse event.

Subjects experienced the following AEs of special interest: bleeding (4.3% vs 3.4%), cardiovascular (0% vs 9.1%), hepatic (3.2% vs 3.4%), renal (1.1% vs 1.1%), thrombotic (0% vs 4.5%), and wound healing (1.1% vs 4.5%). Opioid-related AEs also were experienced by fewer subjects in the meloxicam IV group (48.4% vs 70.5%). The most common AEs typically associated with opioid use were nausea (39.5% vs 59.1%), vomiting (16.1% vs 21.6%), constipation (10.8% vs 12.5%), and dizziness (6.5% vs 5.7%), respectively. No meaningful between-group differences were observed in clinical laboratory tests, including hematology, chemistry, or coagulation parameters.

### Health Care Utilization

With respect to charges associated with hospital stays, the total amount was approximately 10% lower for the meloxicam IV group compared with subjects in the placebo group (Supplemental Digital Content 3, Table S2). The duration of hospital stay was numerically shorter for subjects who received meloxicam IV versus placebo, but the difference was not statistically significant (*P *=* *.4935) (Supplemental Digital Content 3, Table S2). Findings for other resource utilization parameters (all-cause readmissions, emergency department visits due to pain, physician office phone calls due to pain, and skilled nursing facility admission and duration) were lower for the meloxicam IV group, but statistical comparisons were not performed (Supplemental Digital Content 3, Table S2).

## Discussion

Although protocols for pain management after TKA have improved patient outcomes, a substantial proportion of patients still experience residual pain and functional limitations, with residual pain being a major factor in patient dissatisfaction [35–38]. Thus, better pain control has the potential to improve patient satisfaction and functionality. Opioids are frequently used for pain control in patients who undergo TKA (>40% of cases), but there are concerns about the negative consequences of overreliance on these agents, including AEs and the risk of dependence [5, 39]. There are data to suggest that preoperative opioid use is associated with worse patient outcomes after total joint arthroplasty [39]. A meta-analysis of six studies that involved assessment of patient-reported outcomes after TKA demonstrated that preoperative opioid use was linked to significantly worse patient-reported outcome scores relative to nonuse of preoperative opioids [39]. Thus, regimens that reduce the need for perioperative opioids may have potential benefits. For example, effective pain management that minimizes opioid use was shown to improve postoperative rehabilitation and decrease length of stay [40]. In addition, data suggest that NSAID use in patients undergoing orthopedic surgery is associated with reductions in the incidence of postoperative nausea and vomiting, with one meta-analysis demonstrating a decrease of approximately 30% in these AEs when NSAIDs were combined with patient-controlled morphine analgesia [41].

Results of the current study demonstrate that meloxicam IV provides additional pain control when included as part of a perioperative multimodal approach to acute pain management in subjects undergoing TKA. Opioid use was 32% lower in the meloxicam IV group than in the placebo group in the initial 24-hour postoperative period (primary endpoint). Reduction in opioid use was also reported in later time periods (48–72 hours, 0–48 hours, 0–72 hours, and 0 to end of treatment). The finding that meloxicam IV reduces opioid use in the first 24 hours after discharge is notable given the lack of reporting on the effect of opioid prescribing practices after discharge with other pain protocols, including enhanced recovery after surgery (ERAS) protocols [42]. The additional pain control provided by meloxicam IV is evidenced by the longer time to the first use of opioid rescue medication in the active-treatment arm. Furthermore, meloxicam IV was associated with improved pain scores (vs placebo) in the postoperative period. Significant differences were observed in the early postoperative period (first dose to 24 hours) and in later time intervals (ie, first dose to assisted ambulation and first dose to discharge). Improved pain in the immediate postoperative period was also evidenced by improvement in the subject-reported OBAS score on the first postoperative day. The lower pain scores at first assisted ambulation and at discharge, shorter LOS, and the lower use of opioids at discharge suggest that subjects were ready to discharge sooner after receiving meloxicam IV. It is also noteworthy that the better pain scores achieved with meloxicam IV were apparent even without adjusting for increased opioid use in the placebo arm. These results are consistent with those of a post hoc analysis of a phase 3 trial in which meloxicam IV was evaluated in subjects who underwent major surgery: total opioid consumption was substantially lower in the meloxicam IV group than in the placebo group [21]. The effect was most evident among subjects who underwent orthopedic procedures; opioid use was 23.6% lower during treatment with meloxicam IV [21].

This study demonstrates that the number, intensity, and frequency of AEs reported by subjects in the meloxicam IV arm were similar to, or lower than, those reported by subjects in the placebo arm. The overall incidence of AEs did not differ from that of placebo, and there was no indication of an increased risk of events commonly associated with NSAIDs, such as bleeding, wound healing, and cardiovascular, hepatic, renal, or thrombotic events. Decreased opioid consumption in the meloxicam IV group correlated with a reduction of AEs commonly associated with opioids, particularly nausea and vomiting. Two items on the 7-point OBAS questionnaire were “distress and bother from vomiting” and “distress and bother from itching”; thus, reduction in these opioid-related AEs was coincident with the improvement in OBAS scores. In addition, meloxicam IV was associated with a lower cost of hospital stay.

TKA is among the most painful surgical procedures [43], and postoperative pain is a major determinant of delayed discharge after TKA [44, 45]. Findings of the present study are consistent with other data indicating that effective pain control reduces health care resource use for subjects who undergo TKA [46, 47].

The interpretation of the results of this study are limited by the absence of active comparators. This was the first orthopedic study conducted with IV meloxicam initiated preoperatively and in the setting of multimodal therapy; thus, the study was designed to evaluate the efficacy and safety of meloxicam IV in this population in a multimodal setting against placebo. Additional studies including active comparators (eg, oral meloxicam, IV NSAIDS) are needed to determine relative efficacy and safety of these agents in this setting.

## Conclusions

Perioperative administration of meloxicam IV 30 mg as part of a multimodal analgesic regimen demonstrated an opioid-sparing effect among subjects who underwent elective primary TKA. Meloxicam IV had a favorable AE profile comparable to that of placebo and was not associated with an increase in AEs associated with NSAIDs. Select measures of health care resource utilization also tended to be lower with meloxicam IV, including 10% lower mean total hospital charges. These results suggest that meloxicam IV has a promising role in multimodal analgesic regimens in this clinical setting.

## Supplementary Material

pnab016_Supplementary_Data

## Notes

**Funding Statement:** The clinical trial and its publication were supported by Baudax Bio, formerly Recro Pharma, Inc., Malvern, PA, USA.

**DISCLOSURES**

Name: Richard D. Berkowitz, MD

Contribution: This author helped manage subjects, contributed to the acquisition of data, contributed to the study design and its implementation, drafted the article and/or critically reviewed it for important intellectual content and approved the final article.

Name: Richard Steinfeld, MD

Contribution: This author helped manage subjects, contributed to the acquisition of data, contributed to the study design and its implementation, drafted the article and/or critically reviewed it for important intellectual content and approved the final article.

Name: Alexander P. Sah, MD

Contribution: This author helped manage subjects, contributed to the acquisition of data, contributed to the study design and its implementation, drafted the article and/or critically reviewed it for important intellectual content and approved the final article.

Name: Randall J. Mack, BS

Contribution: This author provided guidance on data interpretation, drafted the article and/or critically reviewed it for important intellectual content and approved the final article.

Name: Stewart W. McCallum, MD

Contribution: This author provided guidance on data interpretation, drafted the article and/or critically reviewed it for important intellectual content and approved the final article.

Name: Wei Du, PhD

Contribution: This author helped provide guidance on data analysis and interpretation, contributed to the study design and its implementation, drafted the article and/or critically reviewed it for important intellectual content and approved the final article.

Name: Libby K. Black, PharmD

Contribution: This author provided guidance on data analysis and interpretation, drafted the article and/or critically reviewed it for important intellectual content and approved the final article.

Name: Alex Freyer, PharmD

Contribution: This author provided guidance on data analysis and interpretation, drafted the article and/or critically reviewed it for important intellectual content and approved the final article.

Name: Erin Coyle, BSN

Contribution: This author provided guidance on data analysis and interpretation, drafted the article and/or critically reviewed it for important intellectual content and approved the final article.

**Acknowledgments**

Assistance with manuscript preparation was provided by Bret Fulton, RPh, and Susan Martin, PhD, of The Medicine Group (New Hope, PA).

Trial Registration: ClinicalTrials.gov Identifier: NCT03434275
