# Supplementary material for: Safety and Efficacy of Perioperative Intravenous Meloxicam for Moderate-to-Severe Pain Management in Total Knee Arthroplasty: A Randomized Clinical Trial
Source: Pain Med. 2021 Jan 27;22(6):1261–71. doi: 10.1093/pm/pnab016 (PMC8185557; doi:10.1093/pm/pnab016)
Supplement: pnab016_Supplementary_Data [file pnab016_supplementary_data.docx]

**SUPPLEMENTAL TABLES**

**Supplemental Table 1. Opioid Consumption (IV Morphine Equivalent Dose, mg)**

|  | Meloxicam IV  30 mg | Placebo |  |  |
| --- | --- | --- | --- | --- |
| Parameter, LS mean (SE) | (n = 93) | (n = 88) | Difference | *P* Value |
| Hour 0–24 | 18.9 (1.3) | 27.7 (1.4) | ⇣ 31.7% | < 0.0001 |
| Hour 24–48 | 14.4 (1.1) | 15.9 (1.1) | ⇣ 9.7% | 0.2549 |
| Hour 48–72 | 6.3 (1.1) | 9.3 (1.1) | ⇣ 32.1% | 0.0306 |
| Hour 0–48 | 33.3 (1.9) | 44.0 (2.0) | ⇣ 24.3% | < 0.0001 |
| Hour 0–72 | 39.5 (2.3) | 52.6 (2.4) | ⇣ 25.0% | < 0.0001 |
| Hour 0–EOT | 32.2 (2.3) | 43.5 (2.4) | ⇣ 25.9% | 0.0002 |
| 0–24 h after discharge | 6.2 (0.5) | 7.7 (0.6) | ⇣ 19.1% | 0.0394 |

EOT = end of treatment; IV = intravenous; LS = least squares; SE = standard error.

**Supplemental Table 2. Healthcare Resource Utilization**

|  | Meloxicam IV  30 mg | Placebo |
| --- | --- | --- |
| Parameter, mean (SD) | (n = 93) | (n = 88) |
| Mean length of hospital stay, h (SE)^a^ | 46.1 (1.7) | 49.9 (3.1) |
| All-cause hospital readmissions, n (%) | 1 (1.1) | 3 (3.4) |
| ER visits due to pain, n (%) | 0 | 4 (4.5) |
| Phone calls due to pain, n (%) | 4 (4.3) | 9 (10.2) |
| Skilled nursing facility admission, n (%) | 5 (5.4) | 13 (14.8) |
| Skilled nursing facility duration, days (SD)^b^ | 0.6 (3.1) | 1.9 (5.0) |
| Total physical therapy sessions per subject, n (%) | | |
| 0 | 1 (1.1) | 4 (4.5) |
| 1–5 | 86 (92.5) | 79 (89.8) |
| 6–10 | 6 (6.5) | 4 (4.5) |
| 11–15 | 0 | 1 (1.1) |
| Total hospital charges |  |  |
| Mean (SD) | $56,424 ($29,925) | $62,864 ($45,254) |
| Median | $49,271 | $50,507 |
| Minimum | $26,303 | $12,963 |
| Maximum | $161,022 | $255,070 |

ER = emergency room; SD = standard deviation; SE = standard error.

^a^ *P* = 0.4935; Kaplan-Meier mean value, measured by time (h) from end of surgery to hospital discharge order written.

^b^ From discharge to postoperative day 30.

**Supplemental Table 3. Principal Investigators and Ethics Committee Details, by Study Site**

| **Site** | **Principal Investigator** | **Name/Address of Ethics Committee** |
| --- | --- | --- |
| 001 | Dr. Richard D. Berkowitz | WIRB |
| 002 | Dr. David L. Boyer | WIRB |
| 003 | Dr. Timothy I. Melson | WIRB |
| 004 | Dr. Kipling P. Sharpe | WIRB |
| 005 | Dr. Charles Hall | WIRB |
| 006 | Dr. Trevor Pickering | Baptist Memorial Health Care Corp., Institutional Review Board, 6025 Walnut Grove Rd. Suite 404, Memphis, TN 38120 |
| 007 | Dr. Eugene J. Dabezies | Sacred Heart Health System, Clinical Investigation Review Board, 5151 N 9th Ave, Pensacola, FL 32504 |
| 008 | Dr. Alexander Sah | Washington Hospital IRB, 2500 Mowry Ave, Fremont, CA 94538 |
| 009 | Dr. Richard Steinfeld | WIRB |
| 010 | Dr. Orrin Scott Swayze, formerly Stanley H. Dysart | WIRB |
| 011 | Dr. Kenneth A. Martin | WIRB |
| 012 | Dr. Eric Allan Eifler | WIRB |
| 014 | Dr. Gwo-Chin Lee | WIRB |
| 016 | Dr. William J. Long | WIRB |
| 018 | Dr. Michele R. D’Apuzzo | WIRB |
| 019 | Dr. David B. Hirst | WIRB |
| 020 | Dr. Jeffrey Davis | WIRB |
| 024 | Dr. Paul J. King | WIRB |
| 025 | Dr. Charles G. Haddad | WIRB |

WIRB, Western Institutional Review Board, 1019 39^th^ Avenue SE Suite 120, Puyallup, WA 98374-2115

**SUPPLEMENTAL FIGURE**

**Supplemental Figure 1.** Total opioid consumption in first 24 hours after end of surgery. IVMED indicates IV morphine equivalent dose; LS, least squares.
